# Association of CCR5Δ32 Deletion and Human Cytomegalovirus Infection With Colorectal Cancer in Tunisia

**DOI:** 10.3389/fgene.2021.598635

**Published:** 2021-12-17

**Authors:** Hanen Chelbi, Refka Jelassi, Sarra Belfkih, Amor Ben Amor, Nasreddine Saidi, Hamza Ben Salah, Nabiha Mzoughi, Imen Ben Dhifallah, Nadia Boujelben, Radhia Ammi, Aida Bouratbine, Ines Zidi, Karim Aoun

**Affiliations:** ^1^ Laboratory of Medical Parasitology, Biotechnologies, and Biomolecules, Pasteur Institute of Tunis, Tunis, Tunisia; ^2^ Faculty of Sciences of Bizerte, University of Carthage, Tunis, Tunisia; ^3^ Public Relations Department, Emirates College of Technology, Media College, Abu Dhabi, United Arab Emirates; ^4^ Faculty of Sciences of Tunis, University of Tunis El Manar, Tunis, Tunisia; ^5^ Laboratory of Clinical Virology, Pasteur Institute of Tunis, Tunis, Tunisia; ^6^ Department of Pathology, Salah Azaïez Institute, Tunis, Tunisia; ^7^ Laboratory of Microorganismes and Active Biomolecules, Sciences Faculty of Tunis, University of Tunis El Manar, Tunis, Tunisia; ^8^ External Consultants Service, Pasteur Institute of Tunis, Tunis, Tunisia

**Keywords:** colorectal cancer, human cytomegalovirus, CCR5Δ32, nested PCR, UL55, UL138 genes

## Abstract

**Background and objectives:** Human cytomegalovirus (HCMV) and genetic polymorphisms of the chemokine receptor 5 have been suggested as factors associated with the progression of colorectal cancer (CRC). The aim of the study was to evaluate the associations of both CCR5Δ32 genetic deletion and/or HCMV virus infection with CRC in Tunisia. Materials and methods: The association between HCMV and CRC was validated by Nested PCR technology performed for HCMV and HCMV-specific serum IgG and IgM antibodies were investigated by enzyme-linked immunosorbent assay. Experiments were carried out on 40 tumor and 35 peri-tumor tissues, 100 blood from CRC patients and on 140 blood samples from healthy subjects and finaly serum samples of 80 patients with CRC and 100 healthy individuals. A conventional PCR has been optimized for the detection of CCR5Δ32 in100 CRC patients and 100 healthy subjects. Results: Our results show that HCMV is significantly active in 93% of patients compared to 60% in controls (*p* < 0.0001, OR = 8.85, 95% CI: 3.82 -20.50). Compared to the healthy controls, the titers of IgG and IgM antiCMV antibodies in CRC patients were significantly higher than in healthy subjects (*p* value < 0,0001 for IgG and IgM). Statistical analysis revealed a lack of association between CCR5Δ32 mutation and colorectal cancer (*p* = 0.788, OR = 1.265, 95% CI: 0.228-7.011). Conclusion: our data confirmed that the HCMV infection was related to the development of CRC and that CRC cells may be infected more favorably by HCMV. Given the importance of the CCR5 in inflammation and therefore CRC progression, further studies still needed to evaluate CCR5 role as a potential candidate gene for CRC susceptibility under other polymorphisms.

## Introduction

Colorectal cancer (CRC) is the third commonly diagnosed cancer and the second leading cause of cancer-related deaths worldwide in 2020 (Hyuna et al., 2021). It is a malignant tumor of the lining of the colon or rectum. 60–80% of CRC developed from an adenoma, are called adenocarcinomas. The other CRCs develop directly without being preceded by a detectable benign tumor (Lafay and Ancellin, 2015). Rectal cancer accounts for about 30% of colorectal cancer with more severe clinical symptoms (Dayde et al., 2017). The incidence of CRC is about three times higher in developed countries than in developing countries (Bray et al., 2018). In Tunisia, colorectal cancer ranks first among digestive cancers (Kassab et al., 2013). Calculation of incidence is difficult due to the lack of a national cancer registry. However, the country has three regional registers: North, Center and South (Kassab et al., 2013). Risk factors studies established that CRC is a complex multifactorial disease implicating genetic factors, environmental factors, inflammatory bowel diseases and also the intestinal microbiota.

Recently, many studies focused on the potential effect of human cytomegalovirus (HCMV) infection on the onset and/or progression of CRC. It is a common pathogen responsible for generally asymptomatic and persistent infections in healthy people. It can cause serious illness in the absence of an effective immune response (in immunologically immature and immunocompromised individuals). HCMV belongs to the *betaherpesvirinae* subfamily, whose structure, expression kinetics and persistence of the viral gene throughout the life of its host are characteristic of other herpes viruses (Mocarski and Courcelle, 2001). Like other herpes viruses, the HCMV has the ability to establish latency in different cell types (Alwan et al., 2019). Glycoprotein B (gB) is a type 1 transmembrane protein and represents a highly conserved class III fusion protein found in members of the Herpesviridae family. The gene that codes for gB (gpUL55) is located in the central region of the unique long genes (UL) of the HCMV genome (Pang et al., 2008). After a primary infection, HCMV established a lifelong latent infection in the host. Monocytes (Taylor-Wiedeman et al. JGV 1991) and CD34^+^ progenitor cells (Mendelson et al., 1996) are sites of latency. Epithelial and endothelial cells (Revello and Gerna 2010) are sites of lytic infection as are smooth muscle cells (Tumilowicz et al., 1985) The HCMV coordinates the expression of two viral genes, UL135 and UL138, which play opposite roles in the regulation of viral replication (Umashankar et al., 2014). UL135 promotes reactivation from latency and virus replication, in part, by overcoming the effects of UL138 which suppresses replication (Leng et al., 2017). After reactivation of the virus, it has been demonstrated by Landolfo and his collaborators that gB (UL55) is an essential glycoprotein which plays a crucial role in the lytic infection by HCMV since it participates in viral entry, the transmission of the virus from cell to cell (Arav-Boger et al., 2002) and cell fusion (Landolfo et al., 2003). UL138 is expressed during both latent and lytic infection. In the absence of UL55 and the expression of UL138, the virus is considered as a latent virus. When both genes are expressed, the virus is considered as a lytic virus. Moreover, the presence of HCMV-DNA and HCMV-protein in the CRC tissues suggests a possible correlation between HCMV infection and CRC. Several studies on HCMV infected cell lines have demonstrated its oncomodulator potential (Dimberg et al., 2013). Indeed, reactivation of HCMV in cancer patients has been closely associated with some chemokines and with their receptors including CCR5 (Chemokine Receptor 5) (Cano et al., 2012). It is established that inflammation triggers the reactivation of HCMV and promotes its replication (Cook et al., 2006).

In the present study, the HCMV infection status in CRC patients and healthy subjects was analyzed through the detection of specific HCMV-specific immunoglobulin (Ig)G and IgM in sera. Additionally, HCMV infection in peripheral blood leukocytes (PBLs) and in tumor tissues was detected through a highly sensitive and specific polymerase chain reaction (PCR) targeting UL55 and UL138, genes that are essential for HCMV proliferation and latency, respectively.

CCL5 chemokine, the ligand of CCR5, is a chemoattractant for eosinophils, monocytes and T cell lymphocytes involved in the inflammatory process promoting cancer progression (Charo and Ransohoff, 2006). CCR5 expression in tumor cells and various host cells plays an important role in tumor progression (Umansky et al., 2017). Indeed, it has been reported that CCR5 and CCL5 might have relevant role in the angiogenic mechanism during tumor cells evasion through the recruitment of inflammatory cells, (Umansky et al., 2017).

The human CCR5 gene, located on chromosom arm 3p21.31, is composed of four exons and two introns (Mummidi et al., 1997; Guignard et al., 1998). Many genetic polymorphisms have been reported for CCR5 including rs2227010 (A > G), rs2734648 (T > G), rs1799987 (G > A), rs1799988 (T > G), rs1800023 (G > A), and rs1800024 (C > T) promoter SNPs (Single nucleotide polymorphism) (Liu et al., 2019). Some genetic polymorphisms of the CCR5 gene could modulate its protein expression and could even lead to the disappearance of the receptor from immune cells surfaces. In this context, one polymorphism is of interest: the CCR5Δ32 deletion (rs333). It corresponds to the deletion of 32 nucleotides from the exon 1 of the CCR5 gene. A genetic analysis of the open reading frame (ORF) of CCR5 gene revealed the deletion of 32 base pairs consisting of nucleotides 794 to 825 (Barmania and Pepper, 2013). Deletion involves a reading frame shift mutation with the inclusion of seven new amino acids after amino acid 174 and a stop codon at the amino acid number 182. The mutant allele codes for 215 amino acids instead of 352 (WT-CCR5) (Barmania and Pepper, 2013). Studies have shown that homozygous CCR5Δ32 mutations lead to a complete lack of surface expression of CCR5 and that heterozygotes CCR5 / CCR5Δ32 have significantly lower CCR5 levels than wild-type homozygotes (Cheng et al., 2014).

The objectives of our study were to examine the association of the genetic polymorphism CCR5Δ32, as well as to determine the frequencies of HCMV infection in colorectal cancer in Tunisia.

## Materials and Methods

### Participants

This case-control study enrolled 100 patients with colorectal cancer (CRC) and 140 CRC-free healthy controls. Participants were recruited from Charles Nicole Hospital (Anatomic Pathology and Cytology department), Mongi Slim hospital (surgery) and the Salah Azaiez institute (Department of Pathology) of Tunisia. A questionnaire was prepared in collaboration with the Public Relations Department (Emirates College of Technology) with the aim of taking into account the sociological criteria of patients. From CRC patients, 100 blood samples and 75 tissues (40 Tumors, 35 peri-tumors) were collected. From tissue samples, sixty-two are included in paraffin block (FFPE): thirteen samples from tumor or peri-tumor tissues fresh frozen. For CRC-free healthy controls (with no previous history of cancer disease) 100 blood samples were collected in EDTA tube.

### Ethical Approval

This study was approved by the biomedical ethics committee of the Pasteur Institute of Tunis and the ethics committee of Salah Azaiez Institute of Tunisia. All participants gave their agreement and signed the informed consent.

### DNA Extraction

For each FFPE and fresh samples, DNA was extracted using QIAamp DNA Mini Kit (Qiagen, Germany) according to the manufacturer’s instructions with some modifications as earlier reported by Jelassi et al. (2017). The extraction of genomic DNA from 100 blood samples was carried out according to the protocol of the salting-out technique established by Miller and his collaborators (Miller et al., 1988).

**FIGURE 1:**
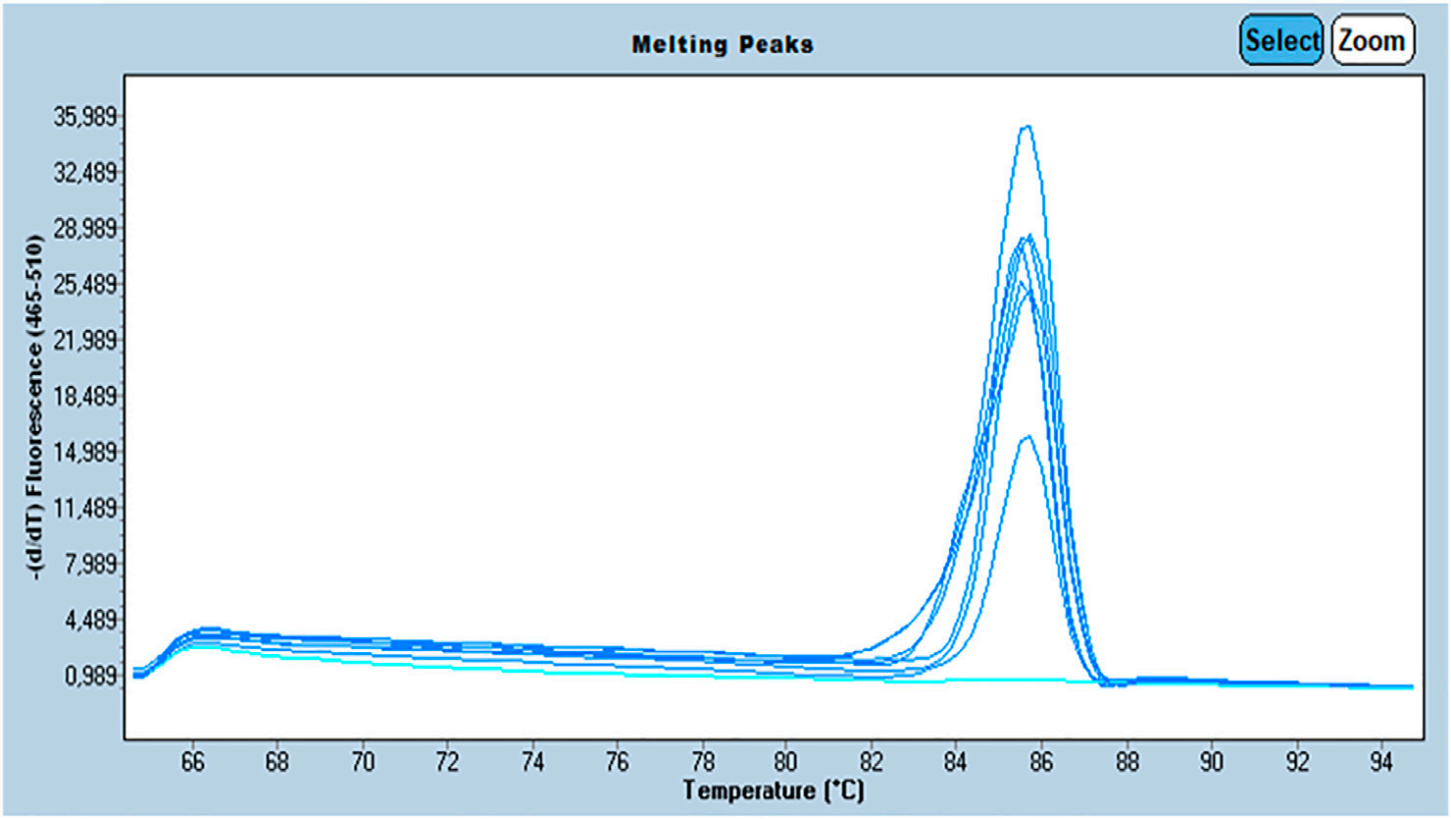
Profile of the melting peaks generated by the amplification *β*-Globin gene by Q-PCR with: The curves in dark blue are the samples positive for *β*-Globin gene and the curves in light blue are the samples negative for *β*-Globin gene.

We assessed the quality of the extracted DNA by amplifying the β-globin gene (internal control) by Q-PCR using BG1/BG2 primers. The primer sequences were as follows: BG1:5’ ACACAACTGTGTTCACTAGC-3’/BG2: 5’ CAA​CTT​CAT​CCA​CGT​TCA​CC-3’ with a target sequence size of 110 bp. All beta globin positive samples underwent further investigation. Amplification of the β-globin was performed on a Roche LightCycler® 480 system (Roche Life Science) using SYBR Green for fluorescence. Melting curves were generated to verify the specificity of the amplification reaction. The presence of a single fusion peak indicates the amplification of a single nucleotide sequence which is verified by gel migration to confirm its size (110 pb) (Figure 1).

### Cytomegalovirus Detection

Amplification of UL55 and UL138 genes was performed by nested PCR using the primers of Jing Chen and his collaborators (Chen et al., 2015). For UL55 gene a 150 bp sequence was first amplified using the stage 1 primers F-5’GAGGACAACGAAATCCTGTTGGGCA3’and R-5’GTCGACGGTGGAGATACTGCTGAGG3’. The PCR product served as a template for amplification of a 100 bp sequence using the stage 2 UL55 primers (F-5’ACCACCGCACTGAGGAATGTCAG3’ and R- 5’TCA​ATC​ATG​CGT​TTG​AAG​AGG​TA3’. For UL138 gene, a 510 bp sequence was first amplified using the UL138 stage 1 primers F-5’ATGGACGATCTGCCGCTGAA3’ and R-5’TCACGTGTATTCTTGATGAT3’. The PCR products served as a template for amplification of an 89 bp sequence using the UL138 stage 2 primers F-5’GCTTACCACTGGCACGACACCT3’ and R-5’TACTCCCCGTACAGCTCGCAAC3’. An adjustment was made to amplify the target sequence of the UL138 gene. One parameter has been optimized: hybridization temperature. All used primers are manufactured by RAN BioLinks, Tunisia. An appropriate negative control (NTC), in which template DNA was replaced by nuclease-free water was run with the samples.

### Testing of HCMV-Specific Serum IgG and IgM

HCMV-specific IgG and IgM antibodies in serum samples from CRC patients and the normal control group were detected by enzyme-linked immunosorbent (ELISA) using an anti-CMV IgG and IgM test kit according to the instructions of the manufacturer (Roche, Mannheim, Germany). The relative levels of antibodies were standardized by known, characterized, positive controls. The ELISA tests were performed using a fully automated ELISA processor (Roche, Mannheim, Germany).

### PCR-Based Genotyping of Allelic Variants of CCR5Δ32 Deletion

The CCR5Δ32 deletion mutation was detected by PCR based techniques as mentioned elsewhere (Mickienė et al., 2014). Tow microliters of extracted DNA was used to amplify a 132-bp-long fragment of *CCR5*, including the deletion with primers 5′-CAC​CTG​CAG​CTC​TCA​TTT​TCC-3′ (forward) and 5′-GTT​TTT​AGG​ATT​CCC​GAG​TAG​CA-3′ (reverse). It is noteworthy that genotypes were detected according to the final size of PCR products, the wild type and the CCR5∆32 genotypes are characterized with a 132 bp and 100 bp amplicons, cycling conditions were 95°C for 5 min, followed by 45 cycles at 95°C for 15 s, 62°C for 30 s, and 72°C for 30 s. A final extension step of 72°C for 5 min was applied. Amplified fragments for the CCR5 locus were resolved in 3.5% agarose gel electrophoresis and visualized by SYBER Safe (Thermo Ficher scientific).

### Statistical Tests

Statistical analyses were performed using GraphPad Prism (Version 9.02, GraphPad software, Inc., United States). The ages among different groups were compared using one-way ANOVA, followed by an LSD test for multiple comparisons correction. Numerical data were represented as *n* (%). The correlation between different groups of detection of HCMV infection was compared using the chi-square test and Fisher’s exact probability. For HCMV serology data were expressed as a median ± standard error of the mean (SEM). The two groups (CRC patients and healthy controls) were compared using the Wilcoxon-Mann-Whitney test. *p* values indicate statistical significance as *p < 0.05, **p < 0.01, ***p < 0.001, and ****p < 0.0001. Exact test for Hardy–Weinberg Equilibrium (HWE) that uses the mid *p*-value was used for rare alleles and low counts (Graffelman and Moreno, 2013) CCR5Δ32 genotyping data analysis was done using the chi-square test and Fisher’s exact probability. *p* < 0.05 was considered significant.

## Results

The demographic and clinical characteristics of both CRC cases and healthy controls are reported in Table 1. There were no statistically significant difference in age and gender between CRC and healthy controls groups. Our study comprised 100 patients with CRC (42 males, 58 females), with mean age of 58 ± 11.46 years, and 140 healthy subjects (66 males, 74 females) with a mean age of 57.3 ± 10.2 years.

**TABLE 1:** Clinical characteristics of patients with CRC and control groups.

Variable	Cases		Controls	
	Tumoral tissues (*N* = 40)	Blood (*N* = 100)	Blood (*N* = 140)	*p* value^a^
Age (mean ± SD)	58 ± 10	58 ± 11.46	57.3 ± 10.2	NS
Sexe male *n* (%)	18 (45)	42 (42)	47 (66)	NS
Cancer site				
Colon (%)	28 (70)	54 (54)	—	—
Rectum (%)	13 (32.5)	35 (35)	—	—
Other (%)	09 (22.5)	10 (10)	—	—
TNM stage				
Satge I	05 (12.5)	09 (09)	—	—
Stage II	12 (30)	34 (34)	—	—
Stage III	09 (22.5)	47 (47)	—	—
Stage IV	14 (35)	10 (10)	—	—
Severity				
Stages I-II	17 (42.5)	47 (47)	—	—
Stages III-IV	23 (63.5)	63 (63)	—	—
Metastases				
Yes	15 (37)	20 (20)	—	—
No	25 (63)	67 (67)	—	—
Not available	0 (0)	13 (13)	—	—

a The age among two groups was compared using one-way ANOVA. NS:not significant.

CRC: colorectal cancer; NS:not significant; SD: standard deviation; N: number.

### PCR Method for Human Cytomegalovirus UL55and UL138 Detection

**FIGURE 2:**
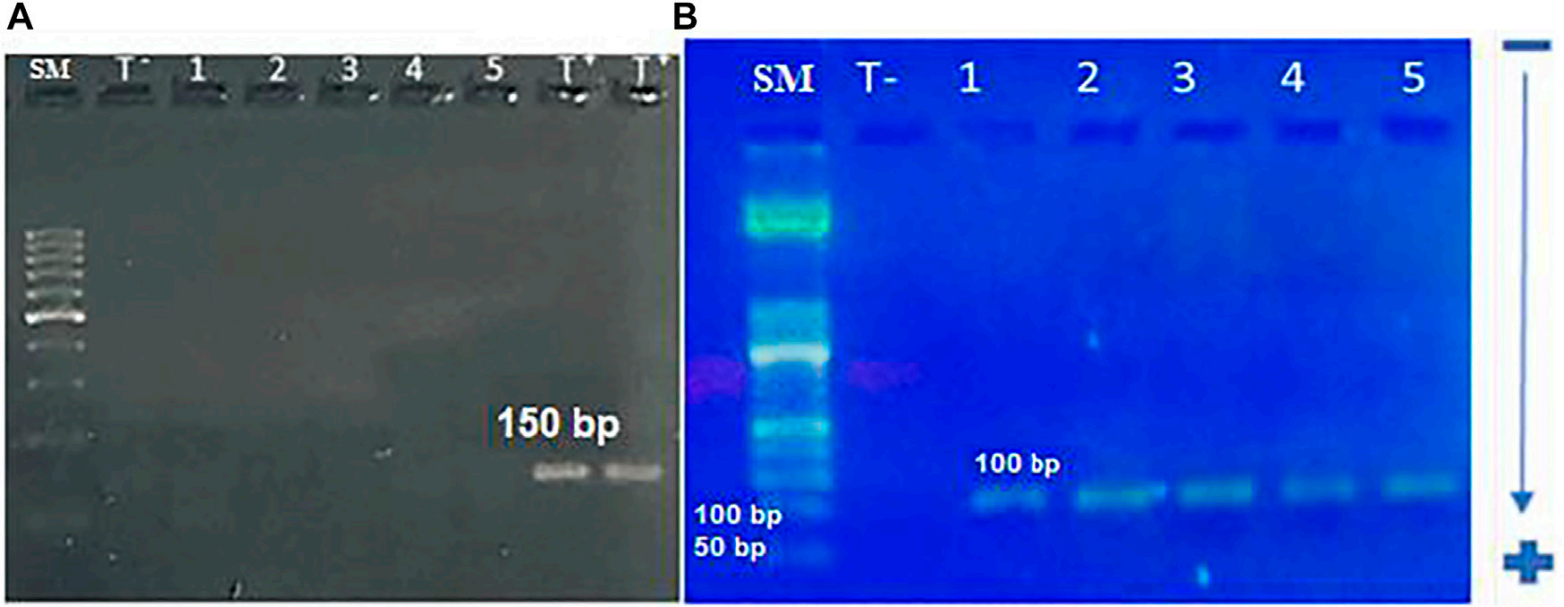
electrophoretic profile of UL55 gene amplification by nested-PCR; **(A)**: Electrophoretic profile of UL55 gene amplification using the first set of primers. A single band is corresponding to the expected size (150 bp).SM: 100 bp size marker, T −: negative control, 1–5: studied samples, T +: positive control **(B)**: electrophoretic profile of UL55 gene amplification using the second set of primers. A single band is corresponding to the expected size of 100 bp. SM: 50 bp size marker, T −: negative control, 1–4: studied samples, 5: positive control.

**FIGURE 3:**
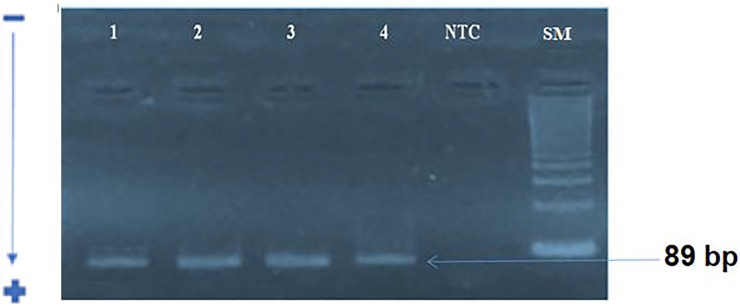
electrophoretic profile of UL138 gene amplification by nested-PCR: A single band corresponding to the expected size of 89 bp for stage 2.SM :100 bp size marker, NTC: Negative control; 1: positive sample; 2–4: studied samples.

We first amplified the UL138 and UL55 genes by nested PCR. The electrophoretic migration of PCR products revealed a single strip in agarose gel corresponding to the expected sizes of 100 bp and 89 bp respectively (Figures 2, 3). CRC frequencies of HCMV-positive samples were calculated for patients and healthy controls. Table 2 showed the prevalence of HCMV in blood samples of both studied groups. Our results revealed a significant difference between the two groups (*p* < 0.0001, OR = 8.85, 95% CI: 3.82-20.50); Table 2). HCMV was positive in 93% of patients and only in 60% for controls (Table 2). Frequencies of HCMV-positive samples calculated for tumor and peri-tumor tissues revealed that HCMV virus was present in its latent form in 100% of the tumor tissues and absent from a single sample of peri-tumor tissues (Table 3). Regarding UL55 gene was more represented in tumor tissues (92.5%) compared to peri-tumor tissues (82.86%) (Table 3). However, statistics do not reach significance for both genes UL55 (*p* = 0.29, OR = 2.55, 95% CI 0.94-1.33) and UL138 (*p* = 0.46, OR = 3.40, 95% IC 0.97-1.09) (Table 3).

**TABLE 2:** Prevalence of HCMV in blood samples collected from CRC patients and controls.

		Cases (*N* = 100)		Control (*N* = 138)		*p* (95% CI)	OR
		*n*	%	*n*	%		
UL55	Positive	93	93	84	60	—	—
	Negative	07	07	54	40	p < 0.0001(3.82—20.50)	8.85
UL138	Positive	98	98	138	98.5	—	—
	Negative	02	02	00	00	0.7344 (0.09—5.12)	0.71

**HCMV:** Human Cytomegalovirus; **CRC**: colorectal cancer; **CI**: confidence interval; **OR**: odd ratio.

**TABLE 3:** Prevalence of HCMV in both types of tissue.

		Tumoral tissue (*N* = 40)		Peri-tumoral tissue (*N* = 35)		*p* (95% CI)	OR
UL55	—	n	%	n	%	0.29 (0.94-1.33)	2.55
—	Positive	37	92.5	29	82.86	—	—
—	Negative	3	7.5	6	17.14	—	—
UL138	—	n	%	n	%	0.46 (0.97-1.09)	3.40
—	Positive	40	100	34	97.14	—	—
—	Negative	0	0	1	2.86	—	—

**HCMV:** Human Cytomegalovirus; **UL**: unique long gene; **CI**: confidence interval; **OR**: odd ratio.

### Human Cytomegalovirus Serology in the Colorectal Cancer Population

**FIGURE 4:**
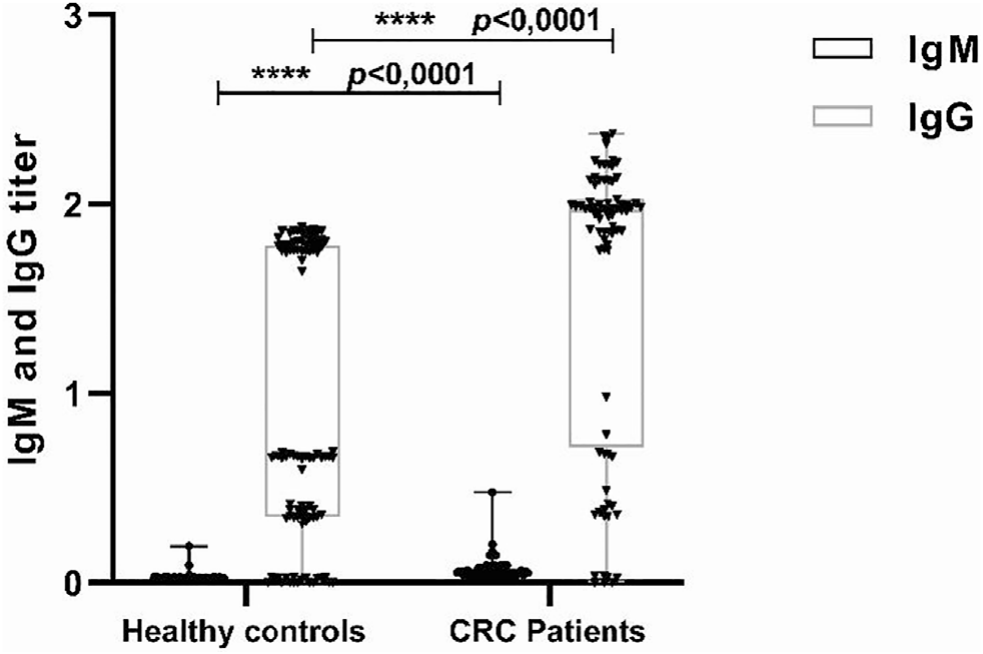
Detection of IgG and IgM antihuman cytomegalovirus (HCMV) in sera from colorectal cancer (CRC) and control patients. Sera were detected by enzyme-linked immunosorbent assay (ELISA) using anti-CMV IgG and IgM test kit. The median of IgG titers to HCMV in CRC patients and control patients were 1.96 versus 0.6710, while the IgM titers to HCMV were 0.019 versus 0.049. ***p = 0.0001, ***p = 0.0001.

To study the impact of the HCMV infection on the CRC, sera from 80 patients with CRC and from 100 control patients were analyzed for the presence of IgG and IgM anti-CMV antibodies. 78 (97.5%) samples from the patient’s group and 56 (56%) samples from the healthy group were positive for anti-CMV IgG. At the same time, there also was no significant difference in the prevalence of IgM anti-CMV antibodies between CRC patients 1 (2.5%) and control patients 2 (2%) (*p* = 0.89). However, the levels of IgG and IgM antiCMV antibodies in the CRC patients were significantly higher than the control patients (*p* = 0.0001 and *p* = 0.0001) (Figure 4).

#### Correlation Between HCMV Antibodies and Presence of Viral Genome

According to 93 PCR HCMV positive cases , (75%) samples were positive for their anti-CMV IgG which was statistically significant (*p* = 0.01). We also, observed no significant result between anti-CMV IgM positivity and genome presence of HCMV in corresponding sample.

### Distribution of CCR5 Allelic Variants

**FIGURE 5:**
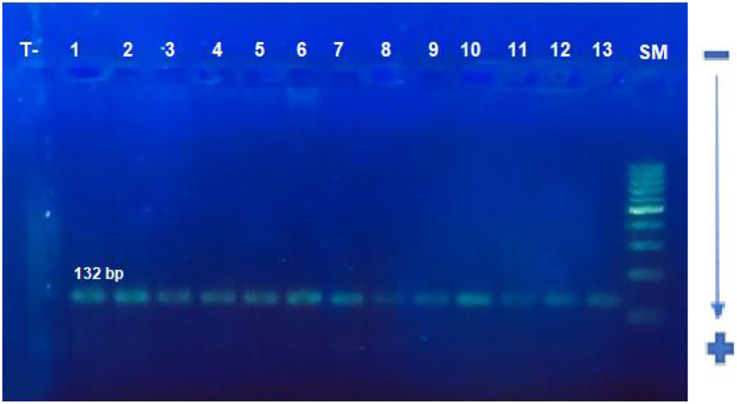
Electrophoretic profile of CCR5 gene containing the CCR5Δ32 deletion using PCR. A single band of 132 bp is corresponding to the size of the target sequence forthe studied samples.SM: 100 bp size, T−: negative control, 1–13: studied samples.

The target sequence of 132 bp was successfully amplified by nested PCR (Figure 5). Exact test for HWE uses the midP-value was performed. This test gives a *p*-value of 0.503 for cases, and 0.515 for controls, showing that equilibrium cannot be rejected for two groups (Table 4). The frequency of CCR5Δ32 mutations in CRC patients and controls is shown in Table 4. One hundred (98%) CRC patients and 140 (97%) healthy controls had CCR5 Wt/Wt homozygosity. Two (2%) of 100 patients had heterozygous and 0 (0%) had homozygous Δ32 mutations; overall 02 (02%) of the HCMV patients had Δ32 mutant alleles (Table 4)**.** On the other hand, none of the healthy controls had a homozygous Δ32mt/Δ32 mt pattern. Five (03%) healthy controls exhibited heterozygosity (Table 4). The association study of CCR5 Δ32 deletion with CRC was assessed under genetic models: Wild type dominant and co-dominant form (Laird and Lange, 2011, chapiter 2). The difference between patients and controls was comparable but not significant (*p* = 0.7) (Table 5). The difference was also not significant with when we compared the allele frequency between studied groups; *p* = 0.47.

**TABLE 4:** Hardy–Weinberg Equilibrium (HWE ) test for case and control groups.

		Case (*N* = 100)		Control (*N* = 140)			
		*n*	%	*p -value* ^a^	*n*	%	*p value* *^a^*
—	Genotypes	—	—	—	—	—	—
—	Wt/Wt	98	98	—	135	97	—
—	Wt/Δ32	2	2	—	05	03	—
CCR5 WT/ Δ32	Δ32/ Δ32	0	0	0.503	0	0	0.515
—	allele	—	—	—	—	—	—
—	Δ32	2	2	—	04	4	—
—	WT	198	98	—	196	96	—

a Exact test for HWE, uses mid-p values was performed because of low counts and rare allele.

**TABLE 5:** study association of CCR5 Δ32 deletion with CRC: a case control study.

	Genotypes	Case (*N* = 100)		Control (*N* = 140)		*p* ^a^ *(IC)*	*OR* ^a^
		*n*	%	*n*	%		
Dominance Δ32>WT	Wt/Wt	98	98	135	97	0.7	1.8
	Wt/ Δ32+Δ32/ Δ32	02	02	05	03	0.37–9.25	
Coodominance WT = Δ32	Wt/Wt	98	98	135	97	0.4757	—
	Wt/Δ32	02	02	05	03	—	—
	Δ32/ Δ32	0	0	0	0	—	—

a Fisher’s exact test was used because of lows sample.

## Discussion

### Human Cytomegalovirus and Colorectal Cancer

The presence of HCMV antigens and nucleic acids in CRC have been determined by molecular and virologic techniques since 1978 and then in 1981 establishing a direct relationship between HCMV virus and cancer initiation (Huang et al., 1978). According the study of Dimberg et al. carried out on Swedish and Vietnamese patients, HCMV’s DNA prevalence significantly differed between cancerous tissues and matched normal tissues suggesting the implication of HCMV in CRC development (Dimberg et al., 2013). However, in other studies, no evidence of a direct association between colorectal cancer and HCMV infection has been found (Akintola-Ogunremi et al., 2005). Our results confirmed the lack of differences between HCMV-positive expression in tumor tissue compared to healthy adjacent tissues (*p* = 0.29, OR = 2.55, 95% CI 0.94-1.33). Indeed, HCMV-positive expression was of 92.5% for tumor tissues and of 82.86% in the peri-tumor tissues. Our results are consistent with the work of Mehrabani-Khasraghi and others in which, out of 15 CRC patients, 8 (53.3%) presented detectable HCMV DNA in their tumor samples, while the normal tissue surrounding the tumor was positive for HCMV DNA in 10 cases (66.7%). In 5 patients with CRC (33.3%), HCMV DNA was found in tumor tissue and matched normal tissue. Therefore, statistical analysis revealed that there was no significant association between the HCMV infection and the CRC (Mehrabani-Khasraghi et al., 2016).

On the other hand, by comparing the prevalence of HCMV in blood samples of CRCpatients and healthy controls, we found a significant association with a value of (*p* < 0.0001, OR = 8.85, 95% CI: 3.82-20.50) since 93% of patients present the HCMV against 60% of healthy controls. To confirm the association between HCMV infection and CRC, HCMV-specific IgG and IgM antibodies in the serum were detected in 80 CRC patients and 100 normal healthy subjects. The detection of the IgG antibodies suggested the presence of HCMV infection, while an increase in IgM antibody levels suggests a primary or reactivation of infection. Our data showed that the frequency of positivity for IgG and IgM antiCMV antibodies was not significantly different between CRC patients and healthy controls, whereas the levels of IgG and IgM anti-CMV antibodies in the CRC patients were significantly higher than those in normal healthy subjects.

These results are consistent with those reported by the meta-analysis carried out in 2016 by Bai and his collaborators (Bai et al., 2016). Human cytomegalovirus (HCMV) has been implicated as a factor that may be associated with the progression of colorectal cancer. According to a 2016 meta-analysis., Data from 4 studies were therefore pooled to provide more reliable evidence. A significant difference was observed in the prevalence of HCMV DNA between cancerous and non-cancerous tissues (OR = 6.59, 95% CI = 4.48–9.69). This observation corroborated the association of HCMV with the formation of colorectal tumors (Bai et al., 2016).

Cancer predisposing risk factors are known to cause cellular injury, which in turn activates normal inflammatory response. HCMV can be reactivated as the latently infected monocytes differentiate into macrophages during migration as a part of this inflammatory response. The classically activated macrophages (M1) carrying a re-activated virus infection, can then infect other cell types, such as fibroblasts, endothelial and epithelial cells, which are more permissive to lytic HCMV infections. HCMV infected cells promote inflammatory and angiogenic secretome, that paracrinally, by intercellular signaling through secretion of cytokines, such as IL-6, TGFβ, GM-CSF and cmvIL-10, induce haemangiogenesis, lymphangiogenesis, cell proliferation as well as immune evasion/immunosupression. HCMV infection in the epithelial cells is evidenced to cause transformation to tumor cells (Kumar et al., 2018). Furthermore It is reported that the HCMV infection contributes to disarm the natural killer cells (NK) and adaptive immune responses. NK cells activation of the cytotoxic T-cell responses displays a crucial function in the cell-mediated first-line host responses against viral infections and cancer initiation (Jost and Altfeld, 2013; Rehermann, 2013; Cerwenka and Lanier, 2016).

The previously recognized human onco-viruses are able to fulfill the first definitions of the hallmarks of cancer, such as essential alterations in the cell physiology, that are required for the cellular transformation. HCMV´s role in tumors has traditionally been depicted as oncomodulatory, i.e. with an ability to affect tumor cells to become more aggressive by enhancing cellular proliferation, survival, immunosuppression, angiogenesis, invasion and by creating a pro-inflammatory environment. This latter role is indeed highly relevant since functions of the tumor micro-environment have recently become recognized as key elements in tumor progression and metastasis in addition to the transforming ability of the virus.

### CCR5 and Colorectal Cancer

CCL5 chemokine as well as its receptor CCR5 have been linked to the promotion of the angiogenesis during tumor cells evasion through the recruitment of inflammatory cells. CCR5 plays a role in regulating inflammation with a great expression on T cells, monocytes, macrophages and dendritic cells. It seems that CCR5 leads to the migration of immune cells to inflamed sites.

The high levels of CCR5 expression have been detected in cancer tissues (Lee and Song, 2015), pushed us to evaluate the implication of the CCR5Δ32 polymorphism in CRC as done for other cancer types including prostate cancer (Balistreri et al., 2009; Kucukgergin et al., 2012a), bladder cancer (Kucukgergin et al., 2012b), gallbladder cancer (Srivastava et al., 2008) and breast cancer (Lee and Song, 2015). In this study, we do not reveal substantial association between the CCR5Δ32 deletion and the CRC. However, given the importance of the CCR5 in inflammation and in CRC progression, it is possible that the CCR5 could be a candidate gene for CRC through other polymorphisms.

### Human Cytomegalovirus CCR5 and Colorectal Cancer

On the other hand we found that patients positive for HCMV in its Lytic form have the CCR5 deletion. a result which needs to be confirmed on a significant size cohort. According to a study by (Corrales et al., 2015), suboptimal expression of CCR5 on HCMV-specific T cells likely results in reduced trafficking of these cells to mucosal and parenchymal tissues, thereby facilitating replication local viral infection and dissemination into the systemic compartment, despite treatment with antivirals. Alternatively, increased expression of CCR5 would promote local inflammatory responses. In turn, inflammation trigger HCMV reactivation and promote HCMV replication (Cook et al., 2006).

## Conclusion

In Conclusion, our result showed significant presence of HCMV genome and anti-CMV IgG in colocrectal cancer patients. Our study introduces last exposure to HCMV as a probable factor that proceeds the development of CRC. The CRC cells may be infected more favorably by HCMV. The data may suggest importance of combination of molecular Prevalence of HCMV in CRC and serologic assessments as a useful tool for better understanding of HCMV contribution in disease.

Finally, in addition to the defined cellular oncogenic changes, the modern, wider concept of hallmarks of cancer brings in the complexity of tumor microenvironment and presence of cancer-causing inflammation, as essential onco-modulatory mechanisms, which relates tumor initiation directly to infections by oncogenic viruses. More research in the field is warranted to substantiate additional links. Molecular factors or mechanisms linking HCMV infection and cancer development need still to be investigated, especially, host chronic inflammatory response, which is a widely recognized basic mechanism for the development of most of infection-related tumors.

The two study populations (Cases and controls) for CCR5Δ32 were in Hardy-Weinberg equilibrium explaining the lack of association of CCR5Δ32 and the occurrence of CRC in Tunisians. It is possible that the CCR5 gene could be a candidate gene for CRC *via* other polymorphisms, hence growing the interest in continuing the analysis of the association of CCR5 genetic polymorphisms and CRC risk.

## Data Availability

The datasets presented in this study can be found in online repositories. The names of the repository/repositories and accession number(s) can be found in the article/Supplementary Material.
